# *In vitro* affinity screening of protein and peptide binders by megavalent bead surface display

**DOI:** 10.1093/protein/gzt039

**Published:** 2013-08-26

**Authors:** Letizia Diamante, Pietro Gatti-Lafranconi, Yolanda Schaerli, Florian Hollfelder

**Affiliations:** Department of Biochemistry, University of Cambridge, 80 Tennis Court Road, CB2 1GA Cambridge, UK

**Keywords:** antibody, directed evolution, emulsion PCR, phage display, protein display

## Abstract

The advent of protein display systems has provided access to tailor-made protein binders by directed evolution. We introduce a new *in vitro* display system, bead surface display (BeSD), in which a gene is mounted on a bead via strong non-covalent (streptavidin/biotin) interactions and the corresponding protein is displayed via a covalent thioether bond on the DNA. In contrast to previous monovalent or low-copy bead display systems, multiple copies of the DNA and the protein or peptide of interest are displayed in defined quantities (up to 10^6^ of each), so that flow cytometry can be used to obtain a measure of binding affinity. The utility of the BeSD in directed evolution is validated by library selections of randomized peptide sequences for binding to the anti-hemagglutinin (HA) antibody that proceed with enrichments in excess of 10^3^ and lead to the isolation of high-affinity HA-tags within one round of flow cytometric screening. On-bead *K*_d_ measurements suggest that the selected tags have affinities in the low nanomolar range. In contrast to other display systems (such as ribosome, mRNA and phage display) that are limited to affinity panning selections, BeSD possesses the ability to screen and rank binders by their affinity *in vitro*, a feature that hitherto has been exclusive to *in vivo* multivalent cell display systems (such as yeast display).

## Introduction

**Fig. 1.**
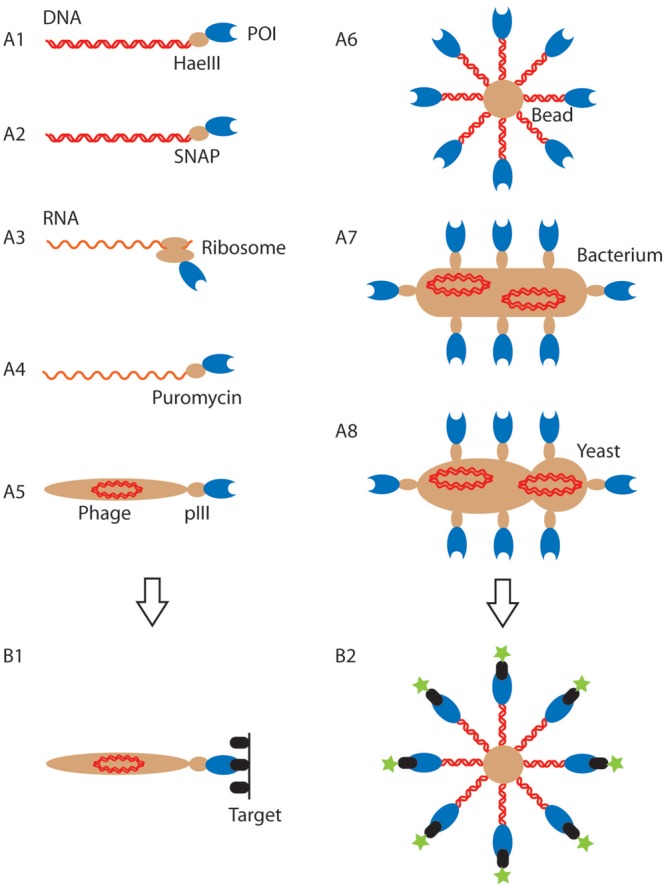
Overview of current display systems. (**A**) Cartoon representation of different genotype–phenotype linkages used in directed evolution (genotype: red; phenotype: blue; entity providing the genotype–phenotype link (protein, ribosome, phage, cell or bead): light brown; the images are not drawn to scale). The specific systems shown are *DNA-display: M-HaeIII* display (A1), SNAP display (A2); *RNA display*: ribosome display (A3), mRNA display (A4); *phage display* (A5). The systems shown in A1–A5 have one copy of the genotype and one or a few copies of the expressed protein. By contrast, *cell-display* methods (bacterial: A7; yeast: A8) have multiple copies of genotype and phenotype. This work describes BeSD (A6), which shares features of both formats, as the displayed protein is expressed *in vitro*, but displayed in up to 10^6^ copies (rather than a single one in other *in vitro* systems), thus endowing BeSD with features that were hitherto exclusive to cell-display systems. (**B**) The display formats imply different selection approaches: panning (shown in B1 for phage display (A5), but carried out analogously for systems A1–4) is based on immobilization of the target on a surface and capture of protein binders by affinity selection. In this process quantitative analysis and direct control of ligand-binding parameters are impossible. Further labor-intensive biophysical measurements are often necessary to assess the strength and specificity of affinity-selected binders. By contrast, flow cytometry (FACS) measures the number of fluorescent target molecules bound directly (B2) and thus *screens* every mutant in the library, allowing a quantitative threshold to be set as the basis for a considered choice during selection. POI, protein or peptide of interest.

High-affinity protein binders with unique specificity have become indispensable reagents in basic research, large-scale proteomic studies and also in therapy, where they represent the fastest-growing segment of the pharmaceutical market. While in nature such binders are generated by the immune system from antibody repertoires, modern display technologies (see Fig. 1 for an overview of existing display constructs) (Leemhuis *et al.*, 2005; Douthwaite and Jackson, 2012) have expanded the range of protein scaffolds used as binders (Gebauer and Skerra, 2009) and enabled better exploration of sequence space. Selections can be performed under *in vitro* conditions, avoiding animal experiments and bias arising from constraints of the host environment (Michnick and Sidhu, 2008; Bradbury *et al.*, 2011). However, protein binders are still not available for all desirable targets and in many instances exhibit imperfect selectivity, lack thermal stability or their suboptimal pharmacokinetic properties necessitate further improvement for clinical applications. The properties of the selected binders are in no small part a function of the selection system used to isolate them, hence, a variety of powerful approaches has been developed.

In the most established technology, phage display, the protein of interest (POI) is fused to a coat protein, e.g. via the N-terminus of the minor (pIII) or major (pVIII) capsid proteins (Fig. 1, A5) (Willats, 2002; Sidhu, 2005; Paschke, 2006). In generating the display construct, the fusion protein is translocated across the *Escherichia coli* cytoplasmic membrane to the periplasm, where it is integrated into the coat of the bacteriophage. Analogous display constructs can be built with bacteria (Francisco *et al.*, 1993; Georgiou *et al.*, 1997; van Bloois *et al.*, 2010) and yeast (Boder and Wittrup, 1997; Gai and Wittrup, 2007) (Fig. 1, A7 and A8). In all of these systems, the POI is covalently linked to proteins on the surface of the organism, and thus indirectly to the genotype as well (as long as the cells do not undergo lysis). Expression occurs *in vivo*, but subsequent selections are carried out *in vitro*.

A number of alternative systems take the expression step into an *in vitro* setting. Ribosome display (Fig. 1, A3) is a non-covalent display system in which the nascent polypeptide chain is coupled to its coding mRNA via the ribosome by deleting a stop codon and avoiding dissociation at high Mg^2+^ concentration and low temperatures (Hanes and Pluckthun, 1997; Dreier and Pluckthun, 2012). Similarly, mRNA display (Fig. 1, A4) relies on connecting genotype and phenotype in the ribosome, although here the bond is covalent via the ribosomal inhibitor puromycin (Roberts and Szostak, 1997; Cho *et al.*, 2000). The benefits of a cell-free format have been demonstrated by comparisons of affinity and diversity of binders generated by ribosome and phage display (Groves *et al.*, 2006; Thom *et al.*, 2006). These quantitative comparisons suggest that the avoidance of the bottlenecks of transformation efficiency and compatibility with cellular machinery improve the success of selections and favor *in vitro* methods. Two conceptually similar *in vitro* systems, *MHaeIII-* (Bertschinger and Neri, 2004; Bertschinger *et al.*, 2007) and SNAP display (Stein *et al.*, 2007; Kaltenbach *et al.*, 2011; Kaltenbach and Hollfelder, 2012; Houlihan *et al.*, 2013) (Fig. 1, A1 and A2) rely on a link between the protein and DNA (instead of the less stable RNA) that is covalent (in contrast to the delicate mRNA–ribosome–polypeptide ternary complex in ribosome display). This linkage is brought about by compartmentalizing a single DNA molecule in each water-in-oil emulsion microdroplet, expressing the POI *in vitro* and retaining both together by the microdroplet boundary. Up to 10^9^ droplets per microliter of aqueous solution can be made by vortexing or using microfluidic devices (Keppler *et al.*, 2003; Courtois *et al.*, 2008; Huebner *et al.*, 2008; Schaerli *et al.*, 2009; Theberge *et al.*, 2010; Devenish *et al.*, 2012; Kaltenbach and Hollfelder, 2012; Kaltenbach *et al.*, 2012). Adjustment of the Poisson distribution ensures that in the majority of occupied droplets only one copy of DNA exists, rendering them ‘monoclonal’. The corresponding protein is expressed as a fusion with a protein tag that reacts covalently with a label on its coding DNA (a modified base (Bertschinger and Neri, 2004; Bertschinger *et al.*, 2007) or a benzylguanine (BG) (Keppler *et al.*, 2003) coupled to DNA).

In addition to the nature of the genotype–phenotype linkage, display systems are distinguished by the way selections are performed (Fig. 1, B1 and B2). Selections on phage-displayed proteins (with typically one or few copies of each variant displayed per phage (Barbas *et al.*, 2004; Clackson and Lowman, 2004) and in current *in vitro* systems are carried out by ‘affinity panning’ based on off-rates (*k*_off_) and therefore highly dependent on the conditions employed (e.g. the duration and number of washes in the panning procedure). Variants are recovered if their affinity is above a pre-set, but not necessarily precisely defined, threshold. When the display constructs contain a larger number of proteins—e.g. ∼10^4^ copies displayed on bacteria (Chen *et al.*, 1996; Andreoni *et al.*, 1997; Christmann *et al.*, 2001; Löfblom *et al.*, 2005, 2007; Rockberg *et al.*, 2008) or 30 000 copies on yeast (Boder and Wittrup, 1997)—selections can be based on the measurement of the binding property of every clone. Here, flow cytometry is employed to rank and sort binders. Variation of the concentration of a fluorescent ligand incubated with the display construct and measurement of the extent to which it, sticks determines selection pressure akin to *K*_d_ titrations. This ranking gives access to populations of weaker and stronger binders depending on the chosen fluorescence threshold in flow cytometry. While ‘panning’ has to be followed up by further labor-intensive biophysical analysis, flow cytometry immediately identifies the best binders in a given sample at high throughput and offers the opportunity to select binders by affinity ranking (based on their *K*_d_).

*In vitro* alternatives to cell-based multivalent display systems would be desirable for selections under conditions that are not compatible with a cellular host, for display of proteins that are toxic and with relative freedom in the size (5PRIME, 2009), and type (Davies *et al.*, 2005) of expressed proteins. The display of nucleic acids and proteins on a bead is the *in vitro* equivalent of such multivalent cell display systems. Initially, single DNA copies were immobilized on beads and droplet compartmentalization used to capture multiple proteins expressed from these templates (Sepp *et al.*, 2002; Griffiths and Tawfik, 2003). Later studies achieved DNA amplification (Gan *et al.*, 2008, 2010; Paul *et al.*, 2013). However, the inefficiency of amplification of bead-bound DNA templates in droplets has in most cases limited this approach to small constructs of <1000 bp (Gan *et al.*, 2008, 2010). The amplification of larger constructs remained unquantified and must be presumed to be inefficient: to the extent that even green fluorescence protein (GFP) could not be detected by its own fluorescence, but required an exhaustively-labeled anti-GFP antibody (Paul *et al.*, 2013). In all these studies, the number of displayed nucleic acids after amplification and the number of displayed proteins also remained undetermined, compromising the quantitative readout on which selection is based. In single DNA bead display (Sepp *et al.*, 2002), hits were detected by tyramide signal amplification, which allows the identification of hits, but not their fine quantitative ranking that is possible, e.g. in yeast display (VanAntwerp and Wittrup, 2000). Furthermore, the use of antibody interactions in building up the display construct limited its stability and thus the robustness of the selection schemes.

In this work, we describe a new type of display construct that presents up to 10^6^ copies of both the DNA template and the encoded protein, each of which can be precisely controlled. Bead surface display (BeSD) combines the advantages of multivalency seen in current cell-based approaches with the potential of *in vitro* methods, while avoiding their respective shortcomings arising from low transformation efficiency (e.g. in yeast display), and lack of display construct stability (e.g. in RNA or ribosome display). The method has been validated by screening libraries of the hemagglutinin (HA)-tag with three randomized positions and successfully isolating the wild-type (WT) HA-tag sequence after a single round of screening. The observation of binding saturation curves (reflecting *K*_d_ values of the isolated variants) of candidates displayed on beads supports the idea that selection is based on direct assessment of the amount of bound ligand. The course of selection during such more informed ‘deep mining’ is thus based on a genuine biophysical measurement, and validation of the hits is possible in the same format. The straightforward protocol and reliable procedures provide a new practical route to expanding the scope of molecular evolution by functional ranking of *in vitro* expressed libraries.

## Materials and methods

### Standard procedures

#### Expression constructs

The plasmid pIVEX-SNAP-HA was derived from pIVEX-SNAP-GFP (Keppler *et al.*, 2003; Mollwitz *et al.*, 2012) by double digestion with NotI and BamHI and subsequent ligation with T4 DNA ligase (1 h, room temperature) to the overlapping oligonucleotides F-HA and R-HA coding for the HA-tag (Supplementary Table S3). F-HA and R-HA were mixed and incubated with a ramp from 85°C to room temperature to let them anneal, before ligation into the digested vector. pIVEX-SNAP-GFP contains the R30I mutant of the SNAP-tag (Sun *et al.*, 2011). The plasmid pIVEX-anchor was derived from pIVEX-SNAP-GFP by double digestion with BglII and BamHI, so that the region consisting of promoter, ribosomal binding site and SNAP-GFP were excised. A restriction digest was followed by blunting of 3′- and 5′-overhangs using T4 DNA polymerase (NEB) and by self-ligation with T4 DNA ligase (1 h at room temperature). The ligated plasmids were transformed into chemically competent TOP10 cells according to the manufacturers' instructions. Plasmids pIVEX-SNAP-GFP and pIVEX-anchor are available via the Addgene repository.

#### HA library construction

HA-NNS libraries (incorporating a degenerate codon in which N stands for an equimolar mixture of all four nucleotides and S for an equimolar mixture of G and C) were created by whole plasmid amplification starting from pIVEX-SNAP-HA as a template using Herculase II Fusion DNA Polymerase (Agilent). The following primer pairs (Supplementary Table S4) were used: F-HA-NNS1 and R-HA-NNS1 for the HA-D7 library, F-HA-NNS2 and R-HA-NNS2 for the HA-Y8A9 library and F-HA-NNS2 and R-HA-NNS3 for the HA-D7Y8A9 library.

#### Preparation of spiking anchors

These were created by standard polymerase chain reaction (PCR) using the vector pIVEX-anchor as a template with the primers F-BB and R2-BG. After PCR purification (QIAquick PCR Purification Kit, Qiagen) the desired number of spiking anchors was incubated with beads.

#### DNA quantification on beads via real-time PCR

Beads were diluted and counted with a hemocytometer (Marienfeld, Superior). Each sample contained 500–2000 beads, 0.8 µM of each primer (F-RT-1 and R-RT-1) and 2X SensiMix SYBR No-ROX Kit (Bioline). The RT-PCR (Corbett Research Rotor-Gene 6000) program started with an initial step of 10 min at 95°C followed by 40 cycles of 95°C for 10 s, 60°C for 10 s and 72°C for 5 s. Reactions were performed at least in duplicate and a standard curve was constructed using known concentrations of template DNA in the range 10^4^–10^9^ DNA copies per reaction. The number of DNA copies per reaction was calculated (using the software accompanying the Rotor-Gene 6000 series) and divided by the number of beads/reaction and by the correction factor 0.3 (fraction of beads bearing DNA out of the total amount of beads, see Supplementary Table S1). The number of anchors immobilized on the beads were quantified in the same way, except that primers F-RT-1 and R2 were used. The quantification of template and anchors from beads recovered after sorting showed no significant differences from the data obtained before *in vitro* expression.

#### Fluorescence imaging

The expression of the SNAP-GFP fusion allowed imaging with a fluorescence microscope (Olympus Bx51) at a 10× enlargement ratio. Fluorescence images (Supplementary Fig. S3) were acquired with an integration interval of 5–10 s, depending on the concentration of expressed protein.

#### Affinity assays on beads

The beads were coated with anchors (Step 4, Fig. 1) and incubated for 1 h in phosphate-buffered saline (PBS) containing skimmed milk (3%, w/v). Then, SNAP-HA was expressed with PURExpress™ (according to the manufacturer's instructions), added to the beads and incubated for 20 min at 37°C. The unbound SNAP-HA was removed by washing the beads (once with PBS containing 0.05% Tween20, then twice with PBS). The beads were incubated with Alexa488-labeled anti-HA antibody (0.1–450 nM). After 30 min of incubation at room temperature, the unbound antibody was removed by washing (once with PBS containing 0.05% Tween20 and once with PBS only). The fluorescence of the beads was analyzed by flow cytometry (Cytek DxP8) and the data are displayed in Fig. 7. The normalized median fluorescence curves were fitted to the Hill equation (with the exponential set to 2) (Goutelle *et al.*, 2008) using Origin Pro 8.

### Protocol for an evolution cycle using BeSD

**Fig. 2.**
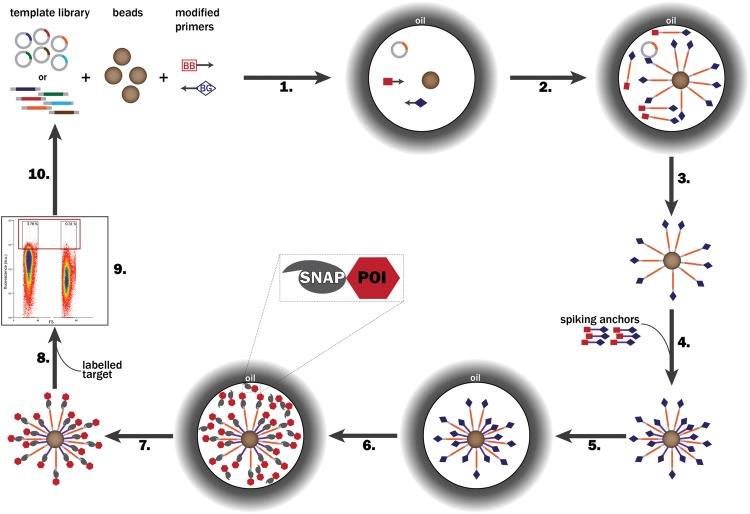
Steps of a directed evolution cycle using BeSD. (1) A DNA library (coding for SNAP-tag-fused POI variants), streptavidin-coated beads and the PCR mix containing BB forward primers and BG-labeled reverse primers are compartmentalized in water-in-oil emulsion droplets so that each compartment contains no more than one DNA template and one bead. (2) DNA is amplified by ePCR and captured on the beads via a biotin–streptavidin linkage. (3) The emulsion is broken, beads washed (to remove PCR mix components and unbound DNA that would compromise IVTT efficiency) and (4) spiking anchors added to provide extra display functionalities. (5) A new emulsion is then created in the presence of an IVTT system. (6) Individual SNAP-tagged POI variants are expressed *in vitro* and covalently linked to the BG-modified template DNA and spiking anchors via the SNAP-tag. (7) After de-emulsification and washing the beads are recovered. (9) The beads are incubated with the labeled target. (9) The affinity for the target is measured by FACS and the selected beads are isolated. (10) The identity of the selected clones is decoded after single-bead PCR by sequencing. Alternatively, the selected beads are used directly for another evolution cycle. The binding affinity of each recovered variant can be measured by subsequent FACS analysis on the bead display construct (see Fig. 6b). POI, protein or peptide of interest.

The following procedure was optimized for ease of handling, robustness and reproducibility. The following steps refer to Fig. 2.

#### Step 1a—Preparation of the PCR reaction

Bioline BioTaq PCR mix (BioTaq buffer (10×), 2 mM MgCl_2_, 0.25 mM of each dNTP and 4.5 U DNA polymerase), 5′-modified biotin-forward primer (F-BB, 0.2 µM), 0.2 µM of BG-modified reverse primer (R2-BG) (prepared as described in (Keppler *et al.*, 2003; Stein *et al.*, 2007; Kaltenbach and Hollfelder, 2012; Kaltenbach *et al.*, 2012; Paul *et al.*, 2013)) or unmodified reverse primer R2 or R3, 1.7 × 10^7^ copies of DNA template (unless otherwise stated in the text) and 9 × 10^5^ streptavidin-coated beads (SiO_2_-MAG-SA-S1964, 5.18 µm, microparticles GmbH) were mixed to give a total volume of 18 µl. Amplification was also possible in the presence of each primer (1 µM) with other polymerases in place of BioTaq, e.g. 2.5× Titanium Taq DNA polymerase (in 1× Titanium Taq PCR buffer; ClonTech), *Pfu* Turbo DNA Polymerase (0.125 U in 1× Cloned *Pfu* DNA polymerase reaction buffer, Agilent).

#### Step 1b—Emulsification

The aqueous phase was mixed with three volumes of an oil phase. The oil phase was composed of the fluorinated surfactant CS99B (a gift from Clive Smith of Sphere Fluidics Ltd and Prof. C. Abell, University of Cambridge) or EA surfactant (a gift from RainDance Technologies) as a 4% (w/w) solution in HFE7500 oil (*n*-C_3_F_7_CF(OC_2_H_5_)CF(CF_3_)_2_, 3M™ NOVEC™) or alternatively in DC749 fluid (30%, w/w; Dow Corning), Triton-100 (1%, w/w) and DC5225C formulation aid (39%, w/w; Dow Corning) in silicone oil (AR 20, Sigma-Aldrich). The emulsion was created by vortexing aqueous and oil phase (in a ratio of 1 : 3) in PCR tubes for 5 min. The emulsions made with the HFE7500 were then pipetted through a 20 µm filter membrane (Celltrics-Partec).

#### Step 2—Temperature cycling

The emulsion PCR (ePCR) temperature program started with a ramp from 25 to 94°C (1°C/s^−1^), followed by 2 min at 94°C and 30 cycles of denaturation (94°C, 30 s), annealing (48°C, 30 s) and extension (72°C, 1 min/kb). After a final extension step (72°C, 5 min), the samples were incubated first at 45°C (5 min) and then at 25°C (20 min) to allow the biotinylated PCR products in solution to attach to the beads.

#### Step 3—De-emulsification

Different de-emulsification procedures were worked out for each oil phase. HFE7500 emulsions were broken by adding water (100 µl, to increase the volume of the aqueous phase for easier handling) and vortexing the samples with 1H,1H,2H,2H-perfluorooctanol (PFO; 200 µl, 97%, Alfa Aesar). The upper phase was transferred in a clean Eppendorf tube. Silicon oil emulsions were broken by adding water-saturated butanol (800 µl). The aqueous and oil phases were separated by spinning for 10 s in a microcentrifuge (Eppendorf). The lower (aqueous) phase was collected. Several repeats of this procedure were sometimes necessary for complete de-emulsification. The beads were washed twice (using a magnet to retain the beads) with deionized water or PBS buffer (pH 7.4, containing 0.05% Tween) and resuspended in deionized water.

#### Step 4—Addition of the spiking anchors

A specific concentration of the anchor DNA (usually 10^7^ anchor molecules/bead) was incubated with the beads, 5 mM Tris/HCl (pH 7.5), 0.5 mM ethylenediaminetetraacetic acid and 1 M NaCl at room temperature for 30 min with shaking (Eppendorf Thermomixer comfort). The non-immobilized spiking anchors were removed by washing the beads twice with water. The number of copies of PCR products and anchors per bead was quantified by real-time PCR (RT-PCR) using primers F-RT-1 and R-RT-1 or F-RT-1 and R2, respectively.

#### Step 5 and 6—*in vitro* expression

*In vitro* transcription and translation (IVTT) reactions were carried out using the PURExpress™ *in vitro* Protein Synthesis Kit (NEB). Reactions of 12.5 µl, consisted of 5 µl of solution A, 3.75 µl of solution B and plasmid or ePCR-amplified DNA on beads in water. For emulsification, the aqueous phase was mixed with three volumes of oil phase (as in Step 1b). The samples were incubated at 37°C for 4–6 h.

#### Step 7—De-emulsification

As in Step 4. The beads were re-suspended in 50 µl of water.

#### Step 8—Addition of the fluorescently labeled target

The beads were incubated with Alexa Fluor^®^ 488-conjugate anti-HA antibody (1 µl; monoclonal mouse IgG1, clone 16B12, Invitrogen) at room temperature for 30 min with shaking. The beads were then washed three times with water or PBS pH 7.5 (300 µl) to remove the unbound antibody before analysis by flow cytometry.

#### Step 9—Fluorescence-activated sorting

Typically, at least 5000 beads were analyzed using a FACScan (Cytek DxP8). Fluorescence-activated sorting was performed with a BeckmanCoulter MoFlo MLS high-speed cell sorter. Beads with fluorescence above a chosen fluorescence value (typically 1% of the population or less) were either individually sorted in 96-well PCR plates (1 bead/well) or pooled in Eppendorf tubes for further use.

#### Step 10—Recovery PCR

Beads sorted by fluorescence-activated cell sorting (FACS) and collected into 96-well plates (one bead/well) were used directly, while pooled samples were diluted to 1 bead/PCR tube and the genotype amplified by PCR using BioTaq or Pfu Turbo DNA Polymerase (Agilent) and primers F-T7 and R-T7 in a standard PCR protocol (performed as in Step 1, but without emulsification) and the amplified products were sequenced.

## Results and discussion

### Assembly of a BeSD construct in microdroplets

Transient compartmentalization of genotype, phenotype and microbeads in an emulsion microdroplet was used to establish multivalent display of *in vitro* expressed proteins (Fig. 1, A6). While compartmentalized in the droplet, single copies of the DNA template are PCR amplified and affinity-captured on the bead. These DNA-displaying beads are de-emulsified and washed, then compartmentalized again to express the POI that is also captured on the bead via the SNAP-tag (Keppler *et al.*, 2003; Stein *et al.*, 2007; Kaltenbach and Hollfelder, 2012; Kaltenbach *et al.*, 2012; Paul *et al.*, 2013). After removal of the droplet boundary, multiple copies of a gene and the corresponding protein are connected on the same bead, together forming a monoclonal and multivalent display construct. Selection cycles and the bioconjugation mechanisms involved are explained in detail in the following paragraphs.

### Display of a protein library on beads for a selection cycle

**Fig. 3.**
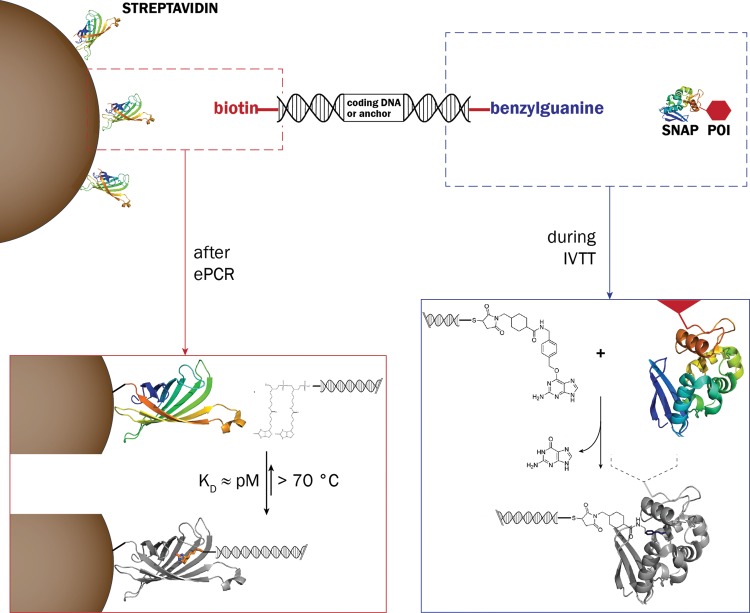
The molecular processes responsible for assembly of the BeSD display construct. Left: DNA molecules are bound to streptavidin-coated beads via biotin. Right: At its other extremity, the DNA molecule carries a BG-label that reacts with an active site cysteine residue of the SNAP-tag (AGT, *O*^6^-alkylguanine-DNA-alkyltransferase) to give a covalent thioether linkage. BG, benzylguanine; IVTT, *in vitro* transcription/translation; POI, protein or peptide of interest.

Figure 2 shows a typical round of BeSD in which a POI library fused to the SNAP-tag is displayed on the beads. First, individual genes are encapsulated in water-in-oil droplets (Step 1) and amplified by ePCR using bis-biotinylated (BB) and BG-modified primers. The amplified DNA is immobilized on the bead via the biotin–streptavidin interaction (Step 2). The emulsion is broken, the PCR reagents removed by washing (Step 3) and, if desired, the beads can be decorated with additional BG-displaying moieties (spiking anchors) in readiness for binding to the POI (Step 4). Protein expression is carried out in newly formed droplet compartments (Step 6) to ensure accurate genotype–phenotype linkage. Following expression, the POIs catalytically link to the beads via covalent coupling of the SNAP-tag to the BG labels (Fig. 3). Finally, FACS can be used to characterize the binding properties of each variant against the desired fluorescent target (Steps 8 and 9). The genotype of each individual bead can be recovered and further analyzed (Step 10). The multivalent genotype leads to better recovery efficiency than do mono- or oligovalent display systems (Supplementary Fig. S1). Furthermore, display of up to 10^6^ proteins per construct enables direct flow cytometric screening, so that a quantitative affinity readout is obtained and the selections are based on a threshold set at will.

### Beads as the centerpiece for multivalent decoration

Commercial streptavidin-coated beads (diameter 5 µm) were employed to provide a support for immobilizing DNA as well as proteins (Fig. 3). The bridging function of the DNA is generated with two types of primers: BB forward primers (to bind to the beads) and BG-modified reverse primers (to form a covalent bond with the expressed protein). The biotin–streptavidin bond is one of the strongest known in biology, with a dissociation constant (*K*_d_) in the order of 4 × 10^−14^M (Green, 1990). However, the *K*_d_ for immobilized streptavidin is reduced compared with streptavidin in solution (Chivers *et al.*, 2010), so BB primers were adopted in an effort to partially compensate for this loss of affinity (Dressman *et al.*, 2003). In order to capture the POI that was *in vitro* expressed from the DNA template, the POI was fused with the SNAP-tag (AGT, *O*^6^-alkylguanine-DNA-alkyltransferase) (Keppler *et al.*, 2003; Mollwitz *et al.*, 2012) that reacts with the BG forming a covalent thioether bond (Fig. 3).

### Tight control of monoclonal compartmentalization

*In vitro* compartmentalization of single genes in water-in-oil emulsion droplets was used as the linchpin for making the bead display construct monoclonal. Emulsion droplets can be easily generated by vortexing a mixture of oil, surfactant and water. For formation of the display construct, a single DNA copy has to be co-compartmentalized with a single bead. The concentration of single components can be adjusted according to the Poisson distribution so that the number of droplets generated exceeds the number of DNA molecules and beads used (Nakano *et al.*, 2003). Supplementary Table S1 shows the probabilities for this desired situation. The correlation between predicted and experimental values was tested by expressing SNAP-GFP in emulsion. A range of template DNA quantities were used for the ePCR step, resulting in different percentages of beads being decorated with the template. Following IVTT in emulsion, the accumulation of fluorescence in droplets can be measured and used as an indicator of GFP expression. Image processing allowed monitoring of the distribution of beads as well as DNA-decorated beads (observed indirectly by the expression of GFP) (Supplementary Table S2 and Fig. S2). Deviations from the theoretical values of bead occupancy (due to the non-homogeneous generation of droplets, bead precipitation, stickiness and stochastic events) are minimized by the distribution of template DNA at the ePCR step. Indeed, protein production can only be observed in droplets containing beads and follows a decrease that is proportional to the amount of DNA template used (55 and 23% for 1.7 × 10^7^ and 4.6 × 10^6^ copies of template DNA, respectively). This experiment can be used to adjust co-encapsulation and monoclonal display.

### Compatibility of emulsions with ePCR and IVTT

The reliability of selections is dependent on the maintenance of compartmentalization during both ePCR at high temperatures and IVTT at 37°C. The stability of the emulsions was thus verified by analysis of images before and after thermal cycling (Supplementary Fig. S3) and after a typical IVTT incubation (4–6 h, Supplementary Fig. S2). No coalescence was observed among >3500 droplets for CS99B/HFE7500 and for EA surfactant/HFE7500. The expression of a SNAP-GFP fusion was used to quantify the expression and the presence of fluorescence in beads-containing droplets (Supplementary Fig. S2, panels A–C) shows that the CS99B/HFE7500 emulsion is compatible with protein expression. The DC surfactants/silicone oil emulsion previously reported (Margulies *et al.*, 2005; Novak *et al.*, 2011) also led to the display of the SNAP-HA fusion on beads (see Supplementary Table S3 and Fig. S4). Efficient *in vitro* expression has also been shown for EA surfactant/HFE7500 (Courtois *et al.*, 2008; Mazutis *et al.*, 2009). The CS99B/HFE7500 emulsion was our preferred combination (Supplementary Table S3), but these data suggest that any biocompatible oil formulation that is able to resist an ePCR cycle can be employed. Compared with the previously published procedures that used mineral oils (Gan *et al.*, 2008, 2010; Paul *et al.*, 2013), the use of *fluorinated* oil/surfactant combination simplifies the handling by allowing rapid, clean de-emulsification with PFO. These surfactants do not interfere with the subsequent steps of the protocol and there is no need for treatment with specific buffers (Dressman *et al.*, 2003; Diehl *et al.*, 2006), the use of water-soluble alcohols (Kumaresan *et al.*, 2008) or mechanical procedures (Margulies *et al.*, 2005). The immiscibility of PFO with water allows for the quantitative recovery of beads in the desired buffer after a simple mixing step.

### Controlled display of multiple DNA copies (genotype amplification)

**Fig. 4.**
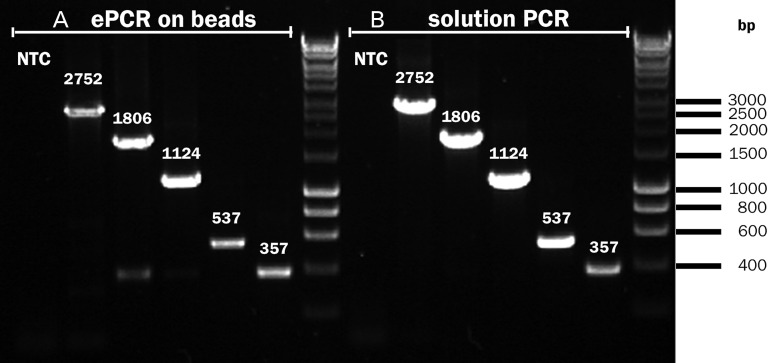
Amplification of bead-bound DNA is possible for templates above 2.7 kb. Amplification was performed (**A**) by ePCR on beads and (**B**) by ordinary solution PCR (in the absence of beads and emulsion). Using the procedure in the Experimental Section (Steps 1–3) it was possible to amplify amplicons ranging from 357 to 2752 bp. After ePCR, the amplicons were purified. A second PCR reaction was used to render the products visible on gel (see Supplementary Table SI for the PCR protocol). Note that this second PCR reaction is not part of the BeSD procedure (shown in Fig. 2 and detailed in the Experimental Section) that only contains a *single* PCR step. See Table I for the quantification of ePCR yields as a function of polymerase species and template length. The initial number of DNA template molecules was 1.7 × 10^7^. The templates were amplified with primers F-BB and R2 (with the exception of the 2752 bp template, where R3 was used instead of R2). NTC, no template control.

It has previously been shown that ePCR on beads is characterized by poor efficiency for amplicons longer than 600 bp (Tiemann-Boege *et al.*, 2009), precluding longer sequence reads (e.g. in 454 sequencing) (Rothberg and Leamon, 2008). By contrast, our ePCR protocol yields up to 10^6^ copies of DNA per bead (as quantified by RT-PCR, see the Experimental Section). Templates as large as 2750 bp could be amplified (Fig. 4, Table I), suggesting that we are able to access larger protein constructs than previous methods. In particular, the addition of an incubation step at room temperature after the PCR leads to a 150-fold increase in the number of DNA copies per bead (Chivers *et al.*, 2010). This step is likely to increase the capture of BB PCR products to the streptavidin-coated beads (Holmberg *et al.*, 2005; Nguyen *et al.*, 2005). Furthermore, a previous protocol employed a covalently bound primer, leading to inefficient ePCR (Paul *et al.*, 2013). In our procedure, there is no need for a preliminary coupling of DNA or primers to the beads and both linear and plasmid DNA can be used as templates for the ePCR. The amplification efficiency was highly dependent on the polymerase employed (summarized in Table I), with *Titanium Taq* giving about 10^3^-fold more product than the proofreading enzyme *Pfu Turbo*.

**Table I.** Amplification efficiencies of different polymerases in ePCR as a function of template length

Amplicon length (bp)	Initial amount of DNA^a^		DNA copies/bead after ePCR^d^		
	Copies/reaction	Copies/bead^b^	TAQ	Titanium Taq	Pfu Turbo
357	1.7 × 10^7^	0.15^c^	1.1 × 10^5^	1.3 × 10^6^	6300^.^
537			2100	1.5 × 10^4^	4^e^
1120			820	5600	8.8^e^
1806			240	8200	4.6^e^
2750			640	1000	n.d.

^a^For a standard reaction (18 µl).

^b^Calculated as the ratio between DNA copies and number of beads (9.0 × 10^5^) corrected for the probability of both beads and DNA being in the same droplet (0.0078, i.e. the sum of ‘desired’ and ‘undesired’ probabilities; see Supplementary Table SI).

^c^This number indicates that, on average, one in every 6.8 beads will bear DNA.

^d^Calculated via RT-PCR on 500 beads per sample. Values represent the average of three measurements and showed standard deviation within 10%, unless otherwise stated.

^e^Standard deviation exceeded 10%.

n.d., not detectable.

### Spiking anchors modulate protein display frequency (phenotype amplification)

**Fig. 5.**
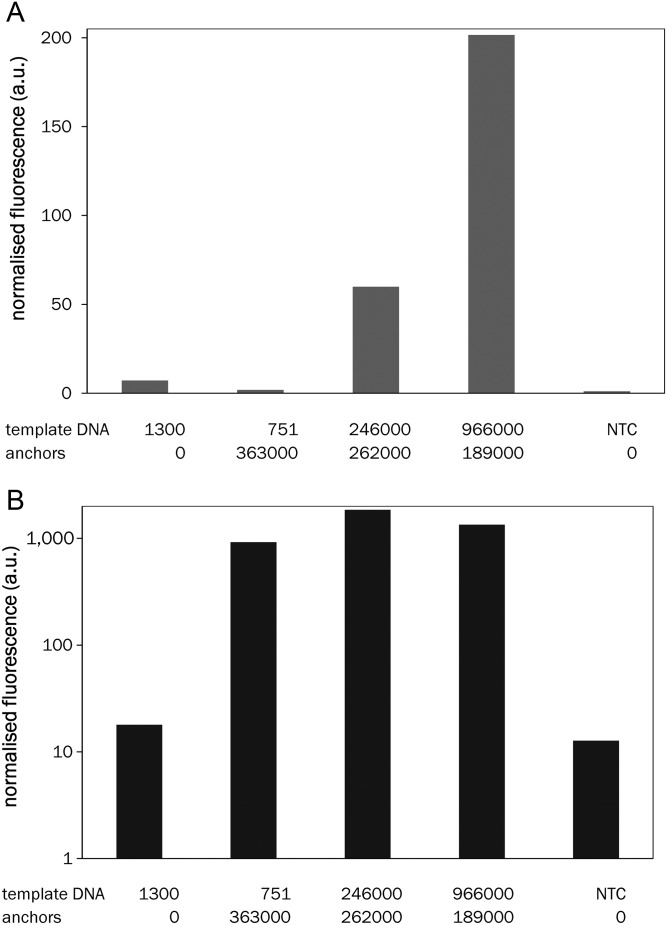
Quantification of the role of spiking anchors in increasing the protein display level. (**A**) Expression of SNAP-GFP from DNA immobilized on bead was measured in the supernatant as a function of the number of DNA templates and anchors displayed on beads (see Supplementary Fig. S5B for a schematic explanation of this experiment). Increasing the expression level (more templates) causes the increase of unbound SNAP-GFP, indicating that all display functionalities on the beads are already saturated under these conditions. DNA concentrations were measured by RT-PCR and have standard deviations below 10%. (NTC, no template control). (**B**) Fluorescence measured on beads after the procedure indicated in Supplementary Fig. S5B. The plot indicates that in the absence of spiking anchors (first set), only a small amount of SNAP-GFP is immobilized on the bead. In the presence of an excess of spiking anchors, however, fluorescence on the beads reaches saturation levels, independently of the amount of template DNA. This suggests that an excess of protein fusion is produced by each template DNA copy and that the display levels reach saturation at low template concentration.

In principle, the efficiency of PCR amplification would create a limit to protein display frequency: each SNAP-tag can react with one molecule of BG only, i.e. the number of displayed molecules cannot exceed the number of successfully amplified genotypes. Limits in the display frequency will reduce the sensitivity of subsequent binding assays. For example, at least 10^3^ copies of the template DNA per bead are required for detection of the expressed SNAP-GFP fusion in flow cytometry (Supplementary Fig. S5A). To provide further valencies for POI capture, free streptavidin moieties on the bead were decorated with ‘*spiking anchors*’ (non-coding DNA bearing BG and bis-biotin labels). Given that tens of thousands of POI molecules are typically produced from each gene by *in vitro* expression (Courtois *et al.*, 2008), the introduction of spiking anchors after PCR amplification enables capture of additional protein copies. Thus, the display frequency can be set independently of the efficiency of the template amplification. The efficacy of *in vitro* expression is dependent on the size of the protein and also on the volume of the droplet compartment, but >30 000 copies per droplet from a single DNA template in solution have been observed (Courtois *et al.*, 2008). Commercial beads (of 5 µm diameter) carry >3 × 10^7^ biotin-binding sites in total, so even relatively inefficient *in vitro* expression (i.e. ∼100 copies per template) should lead to sufficient amounts of protein molecules to decorate *all* spiking anchors, ensuring control over a pre-set, constant display frequency. Given that efficient PCR amplification of larger templates can be difficult to achieve, the ability to uncouple display success from efficient PCR safeguards against a bottleneck is the first step of this procedure. Longer genes or genes with high GC content that amplify less efficiently can thus still be used without compromising protein display frequency. Similarly, the use of proofreading but less efficient polymerases becomes possible. Control over the number of displayed POI copies facilitates subsequent screening by normalizing the number of protein molecules displayed. In yeast and bacterial display dual color selections are used for this purpose (Boder and Wittrup, 1997; Löfblom *et al.*, 2005), while here this level of control is taken care of by addition of a uniform number of anchor molecules (that is larger than the number of PCR products). The total number of BG functionalities per bead can be measured by RT-PCR (by quantifying both the ePCR products and the anchors), allowing precise determination of display levels. To probe experimentally how the number of DNA templates and anchors determined the display level, the amounts of both species to be immobilized on the beads were varied. SNAP-GFP was expressed from these display constructs (with increasing displayed copy numbers of DNA template and with a near-constant number of anchors of around 10^5^). The fluorescence of the supernatant (reporting on the excess of GFP that was *not* captured by the anchors) and on beads (reporting on the GFP captured by the anchors) was measured to quantify when saturation occurs (using a procedure illustrated in Supplementary Fig. S5B). Figure 5A shows that more protein molecules are expressed than display functionalities (template DNA and anchors) are present. This picture is mirrored by the saturation of the GFP fluorescence levels coupled to the beads (Fig. 5B). This type of experiment will be useful to test initially unknown *in vitro* expression and display efficiencies: as long as an increase in bead-bound fluorescence is observed with increasing template or anchor concentrations, the number of display functionalities, rather than expression, limits display levels (i.e. the protein expression is efficient enough to label *all* displayed DNA molecules). Under standard experimental conditions all ∼10^5^ available display functionalities provided by spiking anchors are successfully decorated with expressed protein. To exclude the possibility of exchange of DNA molecules between beads, the stability of the SNAP-HA construct was tested in isolation or mixed with an equal amount of beads that did not display the tag. The fluorescence distribution after 1 h of incubation (i.e. the average time when the beads remain in solution, without compartment separation, during a standard cycle of BeSD) still showed two peaks identical with those of two independent populations, indicating that, at room temperature, no exchange of the displayed proteins occurs (Supplementary Fig. S6).

### Selection of a binding tag from a random library

**Fig. 6.**
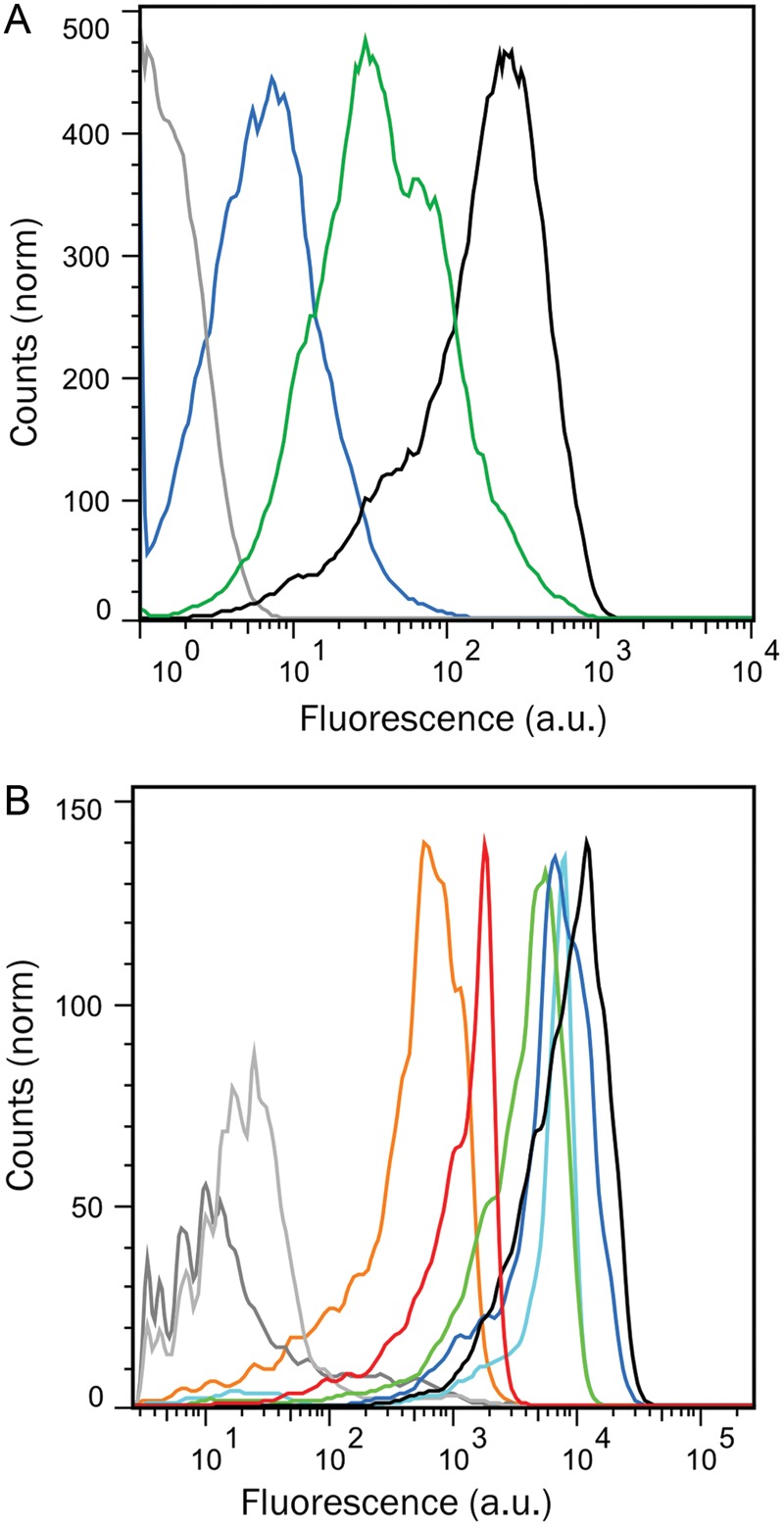
Screening and sorting of the library HA-D7. (**A**) Fluorescence distribution of negative control beads (ePCR without template, gray), beads expressing the SNAP-tag alone (blue), the HA-D7 library (green) or the WT HA-tag (black). (**B**) Characterization of the binding properties of selected mutants by flow cytometry. Genes coding for HA mutants (selected after one round of BeSD) were attached to the beads and Steps 4–9 of the standard BeSD procedure (Fig. 2) were performed but without emulsions. The bead-displayed HA mutants shown are HA-G7 (red), HA-L7 (orange), the selected D7Y8S9 (dark blue) and N7Y8A9 (green) and the designed N7Y8S9 (cyan). Higher fluorescence values indicate greater binding affinity of the HA mutants to Alexa Fluor^®^ 488-conjugate anti-HA antibody (Invitrogen). Controls include beads without template DNA (dark gray), beads displaying the SNAP-tag only (negative control, light gray), and WT HA (black).

To demonstrate the utility of the BeSD method we carried out a selection for a peptide binder from a randomized sequence. A construct was built in which the HA-tag (Field *et al.*, 1988) was fused to the SNAP-tag. The HA-tag sequence (YPYDVPDYASL) was randomized by introducing a degenerate NNS codon in position D7 (library HA-D7). Following the procedure illustrated in Fig. 2, the displayed HA-D7 library was tested for binding against an Alexa488-labeled anti-HA antibody. Flow cytometric analysis showed that the HA-D7 library contained proteins with dramatically different binding properties: one fraction was indistinguishable from the negative control, but there were also binders with WT affinity (Fig. 6A). The ability to recover binders depends on the threshold applied as well as the number of events required for efficient recovery. The top 0.3% of the population were selected (Supplementary Fig. S7), individual beads collected in multiwell plates suitable for PCR, amplified directly and then sequenced. In 86% of the selected population, the amino acid aspartate (that is present in the original HA-tag sequence) had been selected, albeit with alternative codon usage. The remaining clones (14%) contained asparagine. The sequencing of random clones from the unsorted library showed the occurrence of all other amino acids, suggesting that the original library was indeed unbiased. Sequencing of DNA amplified from individual beads confirmed that the first emulsification process (Fig. 2, step 1) leads to monoclonal display constructs (as predicted by the Poisson distribution, Supplementary Table S1). The presence of multiple DNA templates in the same droplet would lead to beads bearing more than one HA variant and generate multiple reads when the corresponding DNA is sequenced. Further evidence for bead monoclonality comes from the observed enrichment values (see below) and from SNAP-GFP expression tests (Supplementary Table S2 and Fig. S2). Should the statistically unlikely multiclonal beads (from the tail of the Poisson distribution) lead to false positives, multiple rounds of sorting can be used to remove them (Kintses *et al.*, 2012).

### Screening of larger libraries and isolation of alternative HA-tags

**Fig. 7.**
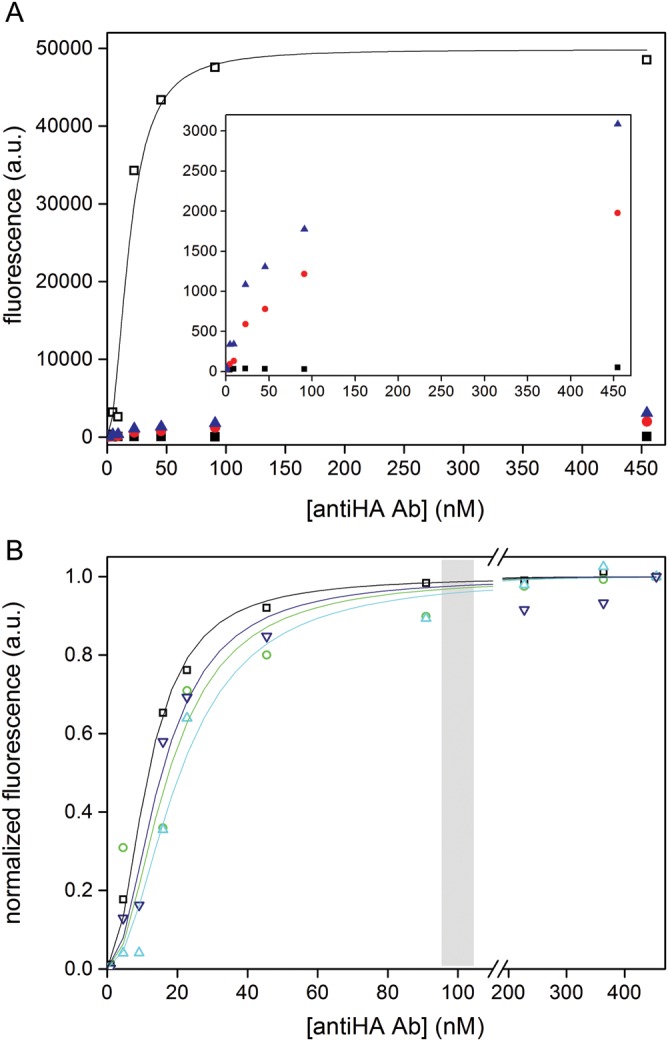
Measurement of binding curves of proteins displayed on the beads by flow cytometry. (**A**) Comparison of WT HA-tag (open squares) with non-binding clones (that were randomly picked from the unsorted library; T7K8L9, red circles and L7Y8A9, blue triangles) and the negative control (beads without DNA, black squares). Inset: enlargement of the same plot excluding the WT data. Note that while the negative control shows a flat line, the two HA-tag variants show an increase in fluorescence that suggests that binding to the anti-HA antibody is occurring and can be quantified (albeit not showing full saturation). (**B**) BeSD display constructs of the WT HA-tag (black squares) and variants isolated from library HA-D7Y8A9 (N7Y8A9, green circles), from library HA-Y8A9 (D7Y8S9, dark blue triangles) and the designed N7Y8S9 (cyan triangles). The beads were incubated with Alexa488-labeled anti-HA antibody (in the range 0.1–450 nM, corresponding to a molar excess of 1–350 times the total number of displayed proteins per sample). A curve fit to the Hill equation gave the following *K*_d_ values: HA-tag WT (11.7 ± 1.1 nM), N7Y8A9 (17.5 ± 1.4 nM), N7Y8S9 (20.7 ± 1.6 nM) and D7Y8S9 (15.5 ± 1.1 nM). The gray box denotes the anti-HA antibody concentrations at which selections were carried out.

Further randomization was undertaken in the HA-tag residues guided by the only available structure of an HA-antibody complex (Churchill *et al.*, 1994). Positions in close contact with the antibody binding site, namely Asp-7, Tyr-8 and Ala-9 of the HA-tag (Supplementary Fig. S8), were randomized. Two libraries containing 400 or 8000 variants were constructed based on full randomization of either the last two (HA-Y8A9) or all three positions (HA-D7Y8A9), respectively. These libraries were sorted by FACS, collecting the top 1.18 and 0.45% of the bead populations (HA-Y8A9 and HA-D7Y8A9 libraries, respectively), followed by DNA amplification for direct sequencing. All of the sequences isolated after one round of screening corresponded to the WT sequence (albeit with alternative codon usages) or mutants in which the character of the WT residues was maintained (e.g. the preference for bulky, hydrophobic residues in position 8 in HA-Y8A9 and the presence of asparagine in position 7 in the HA-D7Y8A9 and HA-D7 libraries, see Table II). The variants selected with higher frequency (D7Y8S9 and N7Y8A9, together with the rationally designed combination between these two N7Y8S9), two variants isolated from gates with lower fluorescence (HA-G7 and HA-L7), and the WT were then expressed *in vitro* (without emulsification) and immobilized on the beads via anchors. FACS measurements of populations of beads displaying these variants (Fig. 6B) indicate that D7Y8S9 and N7Y8A9 possess a fluorescence distribution similar to the WT, as does their combination N7Y8S9. As expected, unselected controls (mutants HA-G7 and HA-L7) have lower mean fluorescence values consistent with reduced affinities for the anti-HA antibody. Thus, fluorescence can be used as a proxy for binding affinity (see also Fig. 7 below). The enrichment in these selections was quantified by comparing the theoretical hit rate and actual recovery. This comparison gave an ideal recovery for the 1-NNS library and half of the ideal recovery for the 3-NNS library (2000-fold).

**Table II.** Sequences of HA variants selected by BeSD

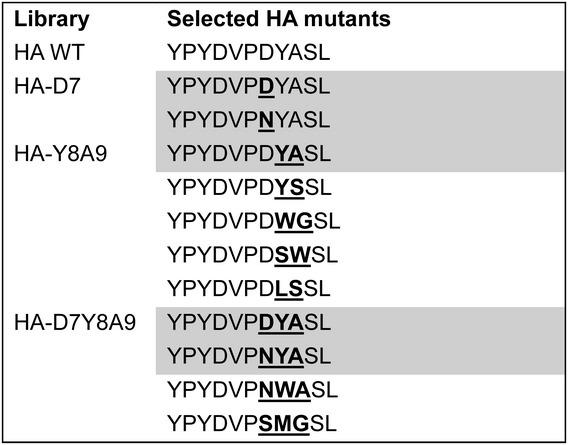

Positions randomized in each library are indicated in bold and underlined. The cell shading highlights mutants that emerged more than once in selections.

### On-bead *K*_d_ measurements

Some of the variants in Fig. 6B were BeSD-displayed, incubated with increasing concentrations of Alexa488-labeled anti-HA antibody and their fluorescence analyzed by flow cytometry. The *K*_d_ was determined by fitting the normalized median fluorescence into a Hill curve (Fig. 7 and supplementary Fig. S9). The curve fit indicates that four variants (HA-tag WT, N7Y8A9, N7Y8S9 and D7Y8S9) possess affinities in the low nanomolar range. The newly isolated HA-tag variants show dissociation constants very similar to that of the WT (the data for N7Y8S8, the weakest binder, can be fit to a *K*_d_ of 20.7 ± 1.6 nM, compared with 11.7 ± 1.1 nM for the WT). Control mutants randomly picked from the unsorted library (HA-L7 and HA-T7K8L9) did not show any saturation even at the highest antibody concentration (450 nM). The correlation between *K*_d_ (Fig. 7) and fluorescence level under screening conditions (Fig. 6B) suggests that in BeSD, the binders are quantitatively ranked and screened on the basis of their affinity for the target. Variants isolated in the same FACS gate (here representing the top 1% of the population, Fig. 6A) differ by as little as 2-fold, suggesting that stringent screening with high resolution is possible. The use of BeSD as a display system combined with flow cytometry offers a quick platform to measure the relative dissociation constant of protein variants. By contrast, affinity quantification is not possible in phage display and other *in vitro* display methods. When bead display systems were applied to selections of binders, the hits were either identified by tyramide signal amplification that does not allow selection for affinity (Sepp *et al.*, 2002) or could not be quantitatively ranked (Gan *et al.*, 2008, 2010). The BeSD thus emulates the hitherto unique ability of cell display systems to carry out affinity selections based on a strict quantitative selection criterion.

## Conclusions

The BeSD differs in several important respects from the currently used display systems. In contrast to bacterial, yeast or phage display, protein expression occurs *in vitro*, so that proteins toxic to the host organism can be displayed. Limits of transformation efficiency (Amstutz *et al.*, 2001) into yeast (∼10^7^–10^8^/µg DNA) and bacteria (∼10^9^–10^10^/µg DNA) that lead to loss of library diversity can be overcome. However, screening remains a bottleneck for the BeSD: the throughput of flow cytometric sorting (FACS) is limited at around 10^8^ clones per day, so that the actual library size probed is more similar to bacteria and yeast display. Similar to these techniques, selections based on fine quantification of the saturation level of a binding curve are possible, allowing deep mining of repertoires of protein binders. The genotype is encoded as DNA (rather than as RNA, as in ribosome and mRNA display) and benefits from a step of amplification via PCR that improves expression and recovery. Other methods relied on just one copy of the gene of interest (Sepp *et al.*, 2002; Griffiths and Tawfik, 2003), which imposes limits on two fronts. On the one hand, recovery of a single gene copy can be challenging, while the availability of multiple templates in the BeSD ensures that all of the selected clones are also identified after PCR and sequencing. On the other hand, more DNA templates lead to more expressed protein molecules, so that the phenotype can be recognized with better sensitivity. Indeed, only one, already very fast enzyme could be evolved in a bead display format (Griffiths and Tawfik, 2003), suggesting that when starting from one gene copy, insufficient protein is expressed to make slower catalysts amenable to evolution. Control over the number of display functionalities on beads can be used further to adjust the selection stringency and normalize display levels, a key feature to avoid bias during the screening step caused by differing expression. This level of control contrasts with previous studies in which the number of displayed proteins was not explicitly quantified or controlled (Gan *et al.*, 2008, 2010; Stapleton and Swartz, 2010; Paul *et al.*, 2013).

Identification of a hit depends on the functional ranking of all of the variants in the library. The high genotype copy number enables DNA recovery from a single bead and provides the possibility to sequence individual variants, without the need for repetitive cycles of enrichment and switching from high- to low- throughput methods. Finally, the display construct is held together by covalent (thioether) and strong, non-covalent and reversible (biotin–streptavidin) interactions, compared with, for example, a potentially unstable adduct in ribosome display. Previously, linkages of the phenotype to beads were achieved via much weaker non-covalent bonds (namely antibodies (Sepp *et al.*, 2002) or the strep-tag (Gan *et al.*, 2010)) and the same functionality was used to bind both the DNA and the displayed proteins. The BeSD relies on conventional techniques (PCR, bulk emulsions and FACS) and on the modular combination of highly controllable steps (decoration of beads, binding stringency and sorting). The potential of the BeSD for the evolution and isolation of new protein binders is based on the following properties:

- *Genotypic redundancy.* Until now, inefficient PCR reactions have prevented decoration of beads with large numbers of copies of coding DNA. The availability of up to 10^6^ templates (depending on the type of polymerase and the length of the gene, Table I) increases the chances of recovery by PCR, ensuring that the hits are identified. Here, the increase in amplification efficiency afforded by slow cooling after the PCR cycles leads to production of more templates than previously possible. Moreover, an accurate quantification of PCR products attached to the beads is achieved by RT-PCR. In previous bead display systems, only one copy was displayed (Sepp *et al.*, 2002; Griffiths and Tawfik, 2003) or the number of amplified templates was not quantified (Gan *et al.*, 2008, 2010; Stapleton and Swartz, 2010; Paul *et al.*, 2013). The amplification and expression of SNAP-HA leads to homogeneous fluorescence signals 20- to 100-fold higher than background noise. Samples that did not undergo ePCR (Supplementary Fig. S1) show a fluorescence signal that is indistinguishable from the background. Incorporation of an ePCR step was therefore essential to increase the dynamic range of the method.
- *Phenotypic redundancy*. Protein expression levels in IVTT can substantially exceed the number of coding DNA molecules (by several orders of magnitude). For example, it has been shown that one copy of template DNA is sufficient to produce >30 000 copies of GFP (Courtois *et al.*, 2008). This quantification suggests that this process can be very efficient indeed. Starting with multiple template molecules in the BeSD is likely to produce even more protein molecule (although it would be optimistic to assume proportionality). It has been shown that a substantial number of a given proteome can be functionally expressed *in vitro* (Davies *et al.*, 2005; Madono *et al.*, 2011), although specific candidates can of course present expression or folding problems, as in any other system. As a high protein display frequency is necessary to carry out sensitive screening assays, a boost in the amount of displayed proteins can be achieved via decoration of the beads with spiking anchors. In this work, we were able to introduce up to 10^6^ spiking anchors via which proteins would be displayed. In cases when it is impossible to generate large numbers of DNA templates by PCR (for example, when use of a proofreading enzyme is required or when the efficiency of the PCR is hampered by difficult templates), this methodology allows the display of a larger number of proteins. At the same time, the ability to control the number of spiking anchors normalizes display levels and neutralizes the effects of stochastic fluctuations in PCR efficiency. In yeast display, the number of displayed molecules is similar to the display level that has been shown to be achieved *in vitro* from one template copy in droplets (Courtois *et al.,* 2008), but is not easily adjustable. In BeSD, the number of displayed molecules can be chosen at will and is pre-determined, so that the quantification of individual expression levels is not required and laborious procedures of double staining (Boder and Wittrup, 1997; Löfblom *et al.*, 2005) can be avoided.
- *The potential for variable DNA and protein display frequency*. The display frequencies can be easily controlled: in the case of template DNA by adjustment of the PCR conditions and in the case of protein display levels by addition of a pre-defined number of spiking anchors (potentially between 0 and 10^6^).
- *Affinity assessment of selected variants on-beads.* While it is not possible to quantify the affinity of proteins displayed on phages or ribosomes, beads combined with flow cytometry offer quick access to relative dissociation constants of the selected variants (reflecting *K*_d_). The same BeSD construct can be used to measure binding saturation curves and thus quantify the affinities of selected mutants.

The ability to display proteins and peptides with a robust and flexible procedure opens up the opportunity to implement directed evolution strategies in which the variants are structurally modified by addition of features designed to improve ‘druggability’ (i.e. protease resistance, serum half-live and pharmacokinetics). The condition for successful chemical modification of a displayed protein prior to selection (‘stapling’) (Verdine and Walensky, 2007; Heinis *et al.*, 2009; Kutchukian *et al.*, 2009) is that the display construct is stable enough and compatible with the chemical reactions performed on it. The stable biotin–streptavidin interaction and the covalent SNAP-tag should fulfill this criterion. Alternatively, non-natural amino acids (*N*-methylated amino acids or unnatural side chains) (Josephson *et al.*, 2005; Goto and Suga, 2012; Hipolito and Suga, 2012), can in principle be incorporated via the BeSD, e.g. when flexizyme (Morimoto *et al.*, 2011) is added to activate a range of non-natural amino acids for *in vitro* incorporation.

Future selection formats based on the BeSD are in principle not limited to selections for protein binders. Selections involving covalent capture of transition state analogs or suicide substrates that lead to covalent capture have already been shown in other display formats. (Amstutz *et al.*, 2002; Cesaro-Tadic *et al.*, 2003; Seelig and Szostak, 2007; Chen *et al.*, 2011) Here, a single turnover marks a library member as a successful catalyst. In the BeSD, the availability of up to 10^6^ valencies allows catalytic efficiency to be measured by a count of the number of turnovers performed in a unit of time over a wide range. As a consequence, the dynamic range of the BeSD should be expanded by orders of magnitude beyond that of monovalent display methods, but future studies will have to show whether this can indeed be experimentally realized.

In summary, the BeSD provides a versatile new tool for directed evolution of functional proteins. Combining robust, reliable and simple procedures, the BeSD should be readily accessible to a wide circle of protein engineers, while at the same time giving access to unprecedented degrees of freedom by combining features of widely used selection formats that are currently mutually exclusive.

## Supplementary data

Supplementary data are available at *PEDS* online.

## Funding

This work was supported by the Engineering and Physical Sciences Research Council, the Biotechnology and Biological Sciences Research Council and the European Research Council (Starting Investigator grant to F.H.), the EU (to L.D. via the Marie-Curie Research Training Networks ProSA and ENEFP and to P.G. via a Marie-Curie fellowship), the Ernst Schering Foundation, the Cambridge Overseas Trust and Trinity Hall, Cambridge (to Y.S.). Funding to pay the Open Access publication charges for this article was provided by BBSRC and EPSRC.

## Supplementary Material

Supplementary Data
