# Supplementary material for: In vitro affinity screening of protein and peptide binders by megavalent bead surface display
Source: Protein Eng Des Sel. 2013 Aug 26;26(10):713–24. doi: 10.1093/protein/gzt039 (PMC3785251; doi:10.1093/protein/gzt039)
Supplement: Supplementary Data [file supp_26_10_713__index.html]

In vitro affinity screening of protein and peptide binders by megavalent bead surface display — Supplementary Data 

# *In vitro* affinity screening of protein and peptide binders by megavalent bead surface display

## Supplementary Data

Supplementary Data

**Files in this Data Supplement:**

- Supplementary Data - Pdf file
