# Supplementary material for: In vitro affinity screening of protein and peptide binders by megavalent bead surface display
Source: Protein Eng Des Sel. 2013 Aug 26;26(10):713–24. doi: 10.1093/protein/gzt039 (PMC3785251; doi:10.1093/protein/gzt039)
Supplement: Supplementary Data [file supp_gzt039_gzt039supp.pdf]

In Vitro Affinity Screening of Protein and Peptide Binders by Megavalent Bead Surface Display (BeSD)

Letizia Diamante,[a] Pietro Gatti-Lafranconi,[a] Yolanda Schaerli,[a] and Florian Hollfelder[a]*

[a] *Department of Biochemistry, University of Cambridge, 80 Tennis Court Road, CB2 1GA, Cambridge (UK)*

* *Corresponding author. E-mail: fh111@cam.ac.uk*

Supplementary Information

Tables

Table S1. Probability values for DNA and bead occupancy in droplets. 2

Table S2. Validation of Poisson distribution of beads in droplets. 3

Table S3. Comparison of the properties of the surfactant systems used in this work. 4

Table S4. Primers used in this work 5

Figures

Figure S1. Dynamic range of SNAP-HA display and effects of ePCR. 6

Figure S2. Assessment of protein expression in bulk droplets after varying the Poisson factor. 7

Figure S3. Emulsion stability under typical ePCR conditions. 8

Figure S4. Display efficiency in different oil/surfactant systems. 8

Figure S5. Analysis of the dependence of BeSD signal on the concentration of spiking anchors. 9

Figure S6. Stability of the display construct. 10

Figure S7. Density plots for WT and HA tag library screening 11

Figure S8. Identification of residues involved in binding of the HA-tag to the anti-HA antibody Fab 26/9. 12

Figure S9. Fluorescence histograms used for the determination of K_d on beads. 13

Additional procedure for Figure 4 14

References 14

Table S1. Probability values for DNA and bead occupancy in droplets.

The table contains the Poisson probabilities[a] for the incorporation of plasmids (first two rows) or beads (third row) in droplets with the indicated average rates of success (μ or Poisson factor). The reported numbers of DNA molecules and beads assume a droplet diameter of 10 μm and an aqueous volume of 18 μl. Rows 4 and 5 contain the combined probability of template and beads distributions. The aggregated probabilities show the percentage of droplets in emulsion that are monoclonal ('desired'), compared to those that will give no phenotype ("irrelevant") and those non-monoclonal ("undesired").

		Number of molecules/beads per emulsion	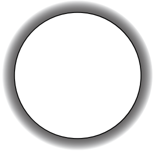	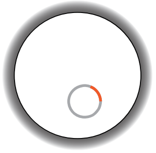	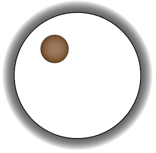	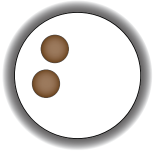	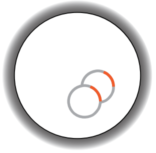	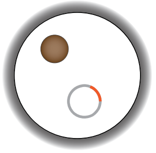	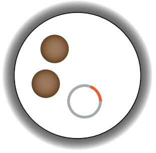	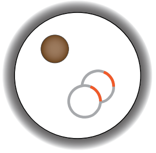	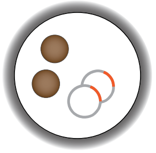
1	pIVEX-SNAP-HA (μ: 0.5)	1.7×10^7	0.606	0.303	0.606	0.606	0.090	0.303	0.303	0.090	0.090
2	pIVEX-SNAP-HA (μ: 0.125)	4.6×10^6	0.882	0.110	0.882	0.882	0.007	0.110	0.110	0.007	0.007
3	beads (μ: 0.02)	9×10^5	0.9802	0.9802	0.0196	$1.97 \cdot 10^{-4}$	0.9802	0.0196	$1.97 \cdot 10^{-4}$	0.0196	$1.97 \cdot 10^{-4}$
4	combined 1 and 3	1.7×10^7 9×10^5	0.594	0.297	0.0119	$1.19 \cdot 10^{-4}$	0.0882	$5.94 \cdot 10^{-3}$	$5.97 \cdot 10^{-5}$	$1.76 \cdot 10^{-3}$	$1.77 \cdot 10^{-5}$
5	combined 2 and 3	4.6×10^6 9×10^5	0.865	0.108	0.0173	$1.74 \cdot 10^{-4}$	$6.86 \cdot 10^{-3}$	$2.16 \cdot 10^{-3}$	$2.17 \cdot 10^{-5}$	$1.37 \cdot 10^{-4}$	$1.38 \cdot 10^{-6}$

			Aggregated probabilities		
		ratio desired/ undesired	irrelevant	desired	undesired
	DNA μ: 0.5	3.4	0.991	0.0060	0.0018
	DNA μ: 0.125	15.7	0.996	0.0022	$1.39\text{-}10^{-4}$

[a] The probability to have x number of plasmids or beads per droplet is given by $P(X=x) = (e^{-\mu}) (\mu^{x})/x!$, where μ is the average number of plasmids or beads per droplet and *e* is the base of the natural logarithm (= 2.7). For a monoclonal droplet x is equal to 1.

Table S2. Validation of Poisson distribution of beads in droplets.

Experimental values of bead and fluorescence distributions obtained from images shown in Figure S4. The numbers in this table are based on data collected from 1200 droplets (with a Poisson factor for the DNA templates of 0.5, middle column) or 840 droplets (with a Poisson factor for the DNA templates 0.125, right column).

	Poisson factor 0.5				Poisson factor 0.125			
	Average number of events	% of the total	% of the subset of droplets containing beads	% of the subset of fluorescent droplets	Average number of events	% of the total	% of the subset of droplets containing beads	% of the subset of fluorescent droplets
Approx number of droplets	1200				840			
Droplets with beads	55.7	5%			48.5	6%		
single beads	47		84.4%		43.5		90%	
multiple beads	8.7		15.6%		5		10%	
Fluorescent droplets	30		54%		11.5		23.7%	
with beads	30			100%	11.5			100%
empty	0			0%	0			0%

Table S3. Comparison of the properties of the surfactant systems used in this work.

	Silicone oil, DC5225, DC749 and Triton 100[a]	HFE7500 and CS99B[b,d] or EA surfactant
**Stability**	Moderately stable at high temperatures	Stable at high temperatures
**Coalescence during ePCR**	Partial coalescence	No coalescence
**IVTT compatibility**	Yes	Yes
**Ease of handling**[c]	Too viscous to filter	Can be filtered
**Ease of de-emulsification**	Hard to demulsify (with water-saturated butanol), so more beads are lost	Very easy to demulsify with 1H,1H,2H,2H-perfluorooctanol, PFO
**Availability**	Commercially available	Commercially available

[a] Silicone oil 30% (w/w), DC5225 39% (w/w), DC749 30% (w/w) and Triton 100 1% (w/w) (Margulies *et al.*, 2005, (Novak *et al.*, 2011)

[b] HFE 7500 with 4% (w/w) fluorinated surfactant CS99B or EA surfactant.

[c] The surfactant systems differ in the filtering step, when emulsions are generated (see Experimental Part). Filtering renders the droplets sample more homogenous in size.

[d] A similar preparation to CS99B is produced by Sphere Fluidics and marketed by Dolomite (Royston, UK) as PicoSurf1.

Table S4. Primers used in this work

All primers were purchased from Invitrogen, except for F-BB which was purchased from IDT.

name	sequence 5' to 3'
F-T7	TAATACGACTCACTATAGGG
R-T7	GCTAGTTATTGCTCAGCGG
R2	AGGGGTTATGCTAGTTATTGCTCAGCGGTG
R2-BG	SH- AGGGGTTATGCTAGTTATTGCTCAGCGGTG
R3	AACGCCTGGTATCTTTATAGTCCTGTCGG
F-RT-1	CGGCGTAGAGGATCGAGA
R-RT-1	CTAGAGGGAAACCGTTGTGG
F-HA	GGCCGCTATCCGTATGATGTACCAGATTATGCAAGCCTCTAATAG
R-HA	GATCCTATTAGAGGCTTGCATAATCTGGTACATCATACGGATAGC
F-HA-NNS1	GGCCGCTATCCGTATGATGTACCANNSTATGCAAGCCTCTAATAG
R-HA-NNS1	GATCCTATTAGAGGCTTGCATASNNTGGTACATCATACGGATAGC
F-HA-NNS2	SNNSAGTCTCTAATAGGATCCGGCTGC
R-HA-NNS2	NNATCTGGTACATCATACGGATAGCGGC
R-HA-NNS3	NNSNNTGGTACATCATACGGATAGCGGC
F-RT-endAGT	GCAATCCTGTCCCTATCCTG
F-BB	/5' DUAL BIOTIN /TTTTTTTTTATGTGCTGCAAGGCGATTAAG

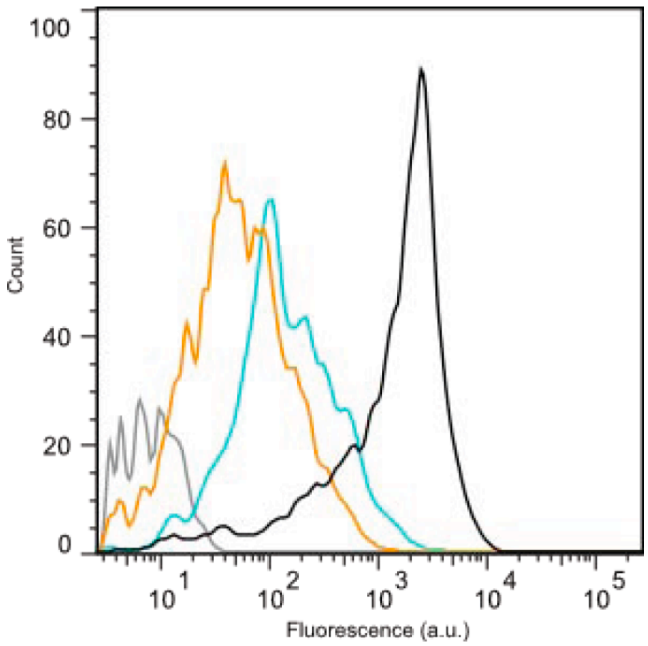

Figure S1. *Dynamic range of SNAP-HA display and effects of ePCR.* FACS analysis of untreated beads (without incubation with fluorescently labelled anti-HA antibody, grey), beads with no DNA attached (cyan), beads bearing only one copy of DNA template (orange) or beads that underwent ePCR (black). The fluorescence signal on beads is increased by almost two orders of magnitude by ePCR, providing strong evidence that amplification of the template by ePCR substantially increase the SNAP-HA expression level, increasing the dynamic range of the method.

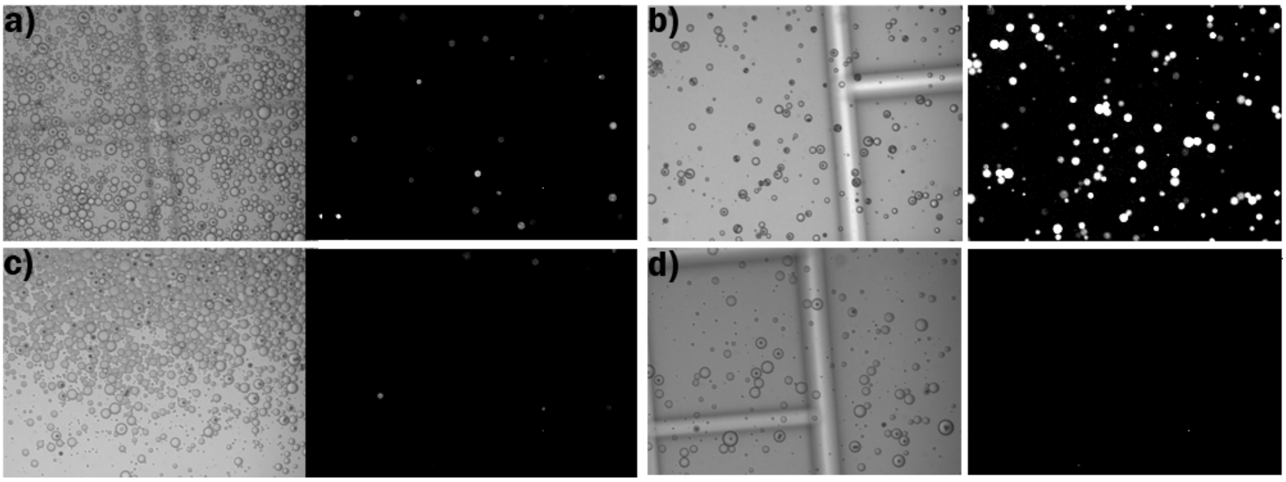

Figure S2. *Assessment of protein expression in bulk droplets after varying the Poisson factor.* Transmission (left) and fluorescence (right) microscopy images after 6 h expression of a SNAP-GFP fusion protein. Panel a): IVTT after ePCR containing 1.7 x 10^7 copies of DNA template (Poisson factor μ=0.5) and 9.0 x 10^5 beads per PCR reaction. Panel b): IVTT with 5.3 x 10^{10} copies (125 ng) of linear DNA and 9.0 x 10^5 beads (positive control). Panel c): same as Panel a but 4.6·10^6 copies of DNA template per PCR reaction (μ: 0.125). Panel d): IVTT from beads that underwent ePCR in the absence of template DNA (negative control). When bead-displayed DNA templates are employed as templates for IVTT, fluorescence is visible only in droplets that contain beads (panels a and c). Decreasing the concentration of initial DNA lowers the number of beads carrying DNA (and consequently the number of beads that yield a fluorescence signal) from 55% to 23% (in panels a and c, respectively). *Conditions:* Emulsification with CS99B/HFE7500, *in vitro* expression with PURExpress (see Experimental Part) at 37 °C. Data show that GFP can be expressed in emulsion with IVTT and that only droplets containing beads are fluorescent. The number of fluorescent droplets can be correlated with expected Poisson probabilities: Table S2 contains the experimentally measured values.

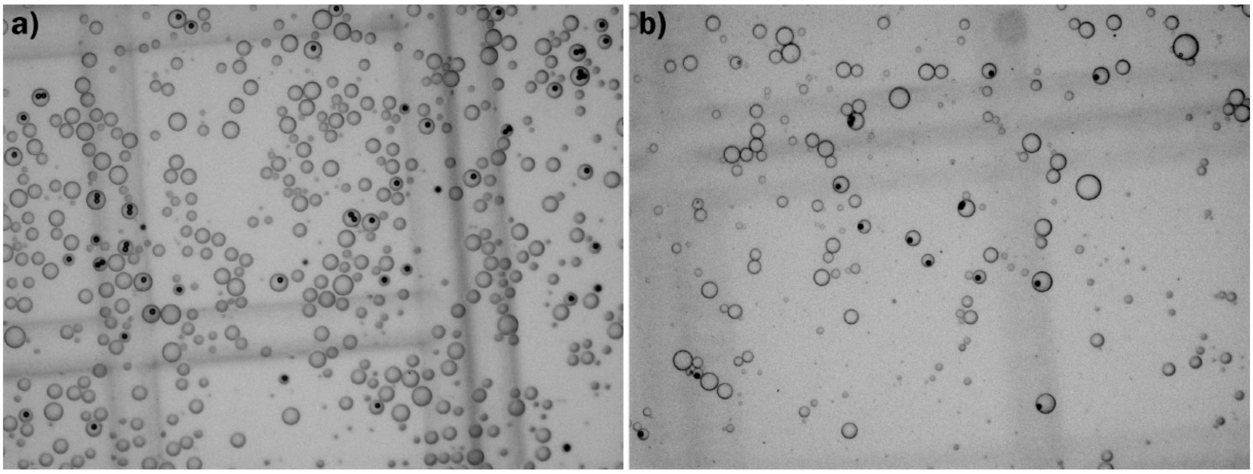

Figure S3. *Emulsion stability under typical ePCR conditions.* An emulsion formed from CS99B/HFE7500 remains stable throughout during ePCR. Brightfield microscopy images (taken with an Olympus BX51 microscope) are shown before (a) and after (b) ePCR. Beads are visible in emulsion droplets as dark spheres. Analysis of 3000 or 1000 droplets before and after ePCR (left and right hand side images, respectively) shows that 10% of them contain at least one bead. Of the latter 20% and 9%, respectively, contain more than one bead (see Table 2).

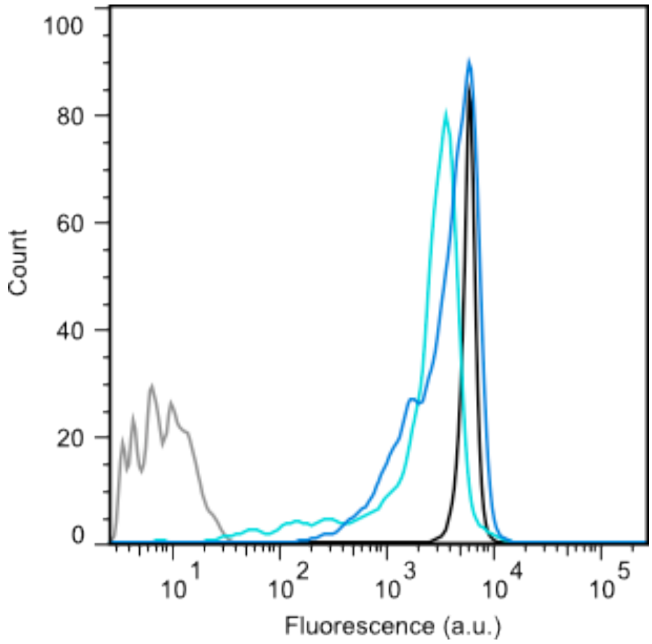

Figure S4. *Display efficiency in different oil/surfactant systems.* Beads were decorated with an identical amount of the SNAP-HA-encoding template and the expression efficiency was measured by flow cytometry after incubation with Alexa Fluor® 488-conjugate anti-HA antibody to assess the following scenarios: (i) expression in the absence of an emulsification step (black), (ii) expression in emulsion compartments made with CS99B/HFE7500 (dark blue) or (iii) emulsion compartments made with DC5225-DC749-Triton 100/silicon oil (cyan). Negative control: Untreated beads (without incubation with the antibody) in grey. Expression was successful in both oil/surfactant systems.

A

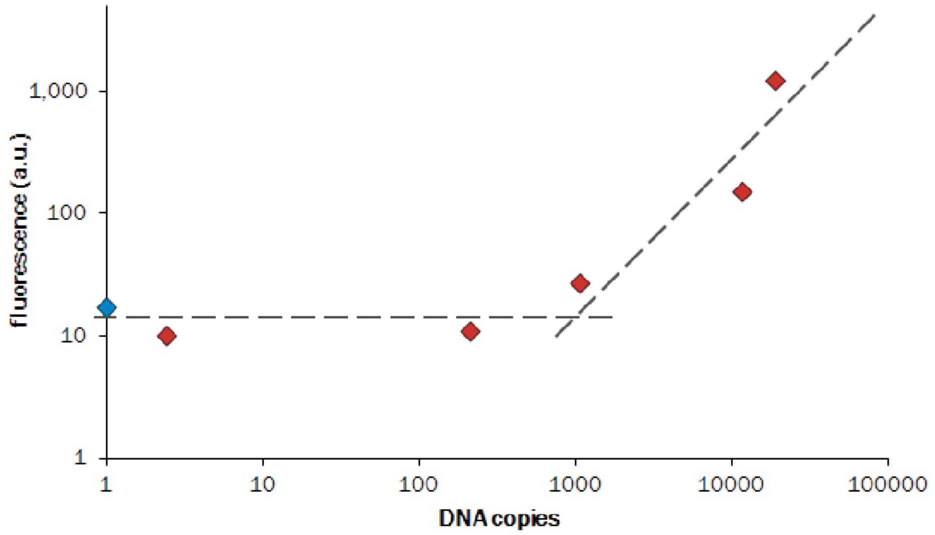

B

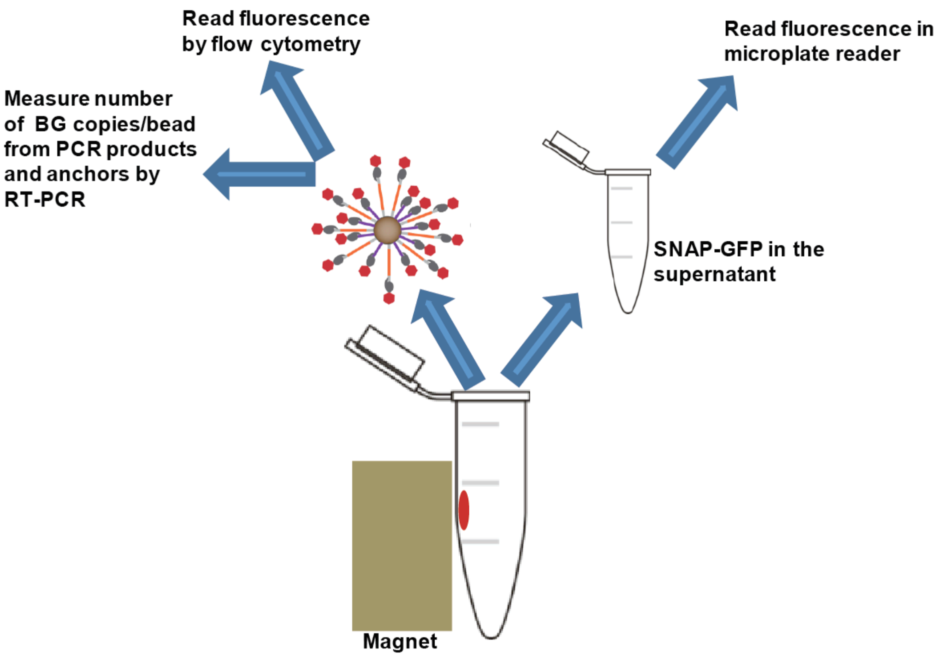

Figure S5. *Analysis of the dependence of BeSD signal on the concentration of spiking anchors.* **(A)** Median fluorescence recorded for *in vitro* expression of a SNAP-GFP fusion protein from defined amounts of template DNA. The template DNA (coding for SNAP-GFP) was immobilized onto beads and contained a BG label. No spiking anchors were used in this experiment, so the number of DNA copies corresponds to the total number of BG molecules displayed on bead. DNA and anchor concentrations were measured by RT-PCR and have standard deviations below 10%. **(B)** Schematic representation of the experiment of Figure 5.

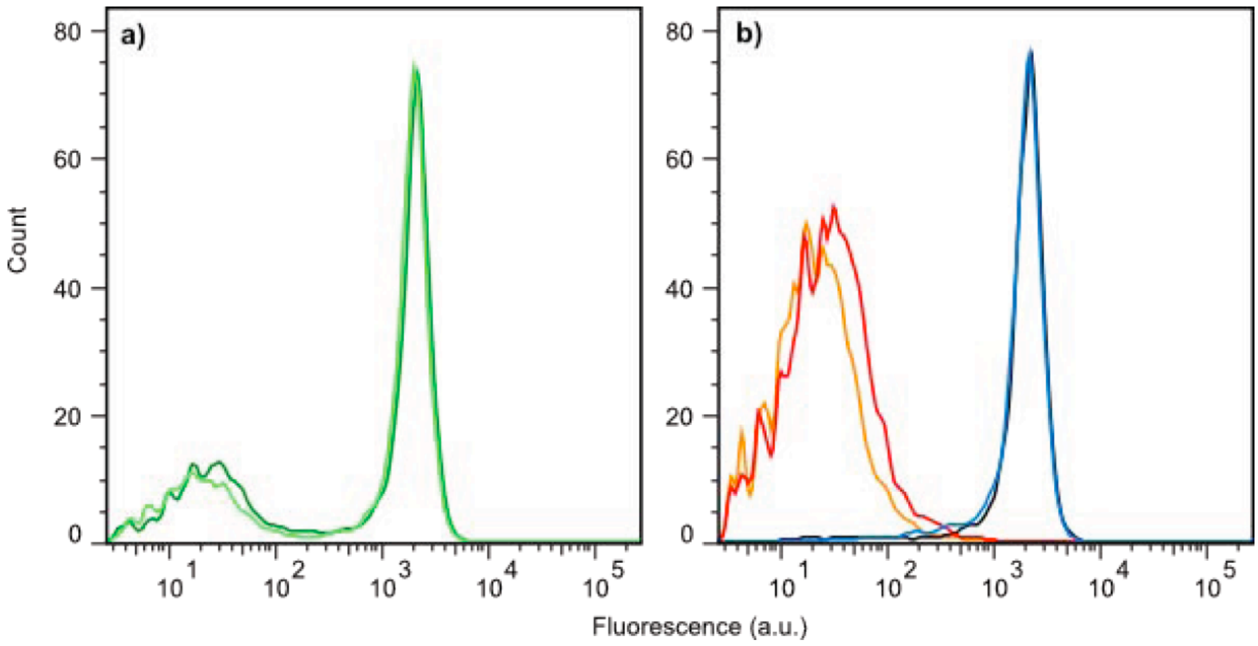

Figure S6. *Stability of the display construct.* Displayed proteins do not exchange on the reaction timescale. Flow cytometry distribution of beads (decorated with spiking anchors) that underwent IVTT in the presence or absence of coding DNA. Peaks with high fluorescence values correspond to beads displaying the SNAP-HA construct, while the lower-fluorescence peak derives from beads that do not display any protein. (a) Fluorescence distribution of a sample containing a 1:1 mix of the two populations measured before (dark green) and after a 1 h incubation in water (light green) shows no cross-contamination between beads. Cross-contamination would result in the appearance of a single peak with averaged fluorescence and the complete absence of such a third peak is strong evidence for stability of the display construct. (b) Independently measured IVTT samples with (black) and without DNA (red) are also stable after 1 hour of incubation (blue and orange, respectively).

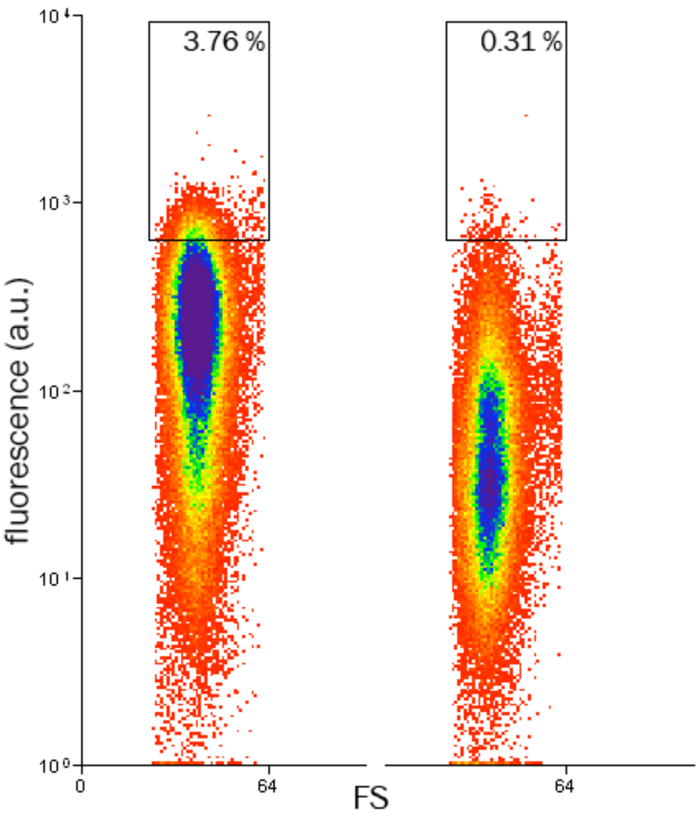

Figure S7. *Density plots for WT and HA tag library screening*. Flow cytometry density plots for WT and HA tag libraries (left and right, respectively) with the position of the sorting gate used indicated by a box. The gate was set to minimize the number of false positives under the conditions of the respective FACS experiments. The percentage of events falling within the gate is annotated.

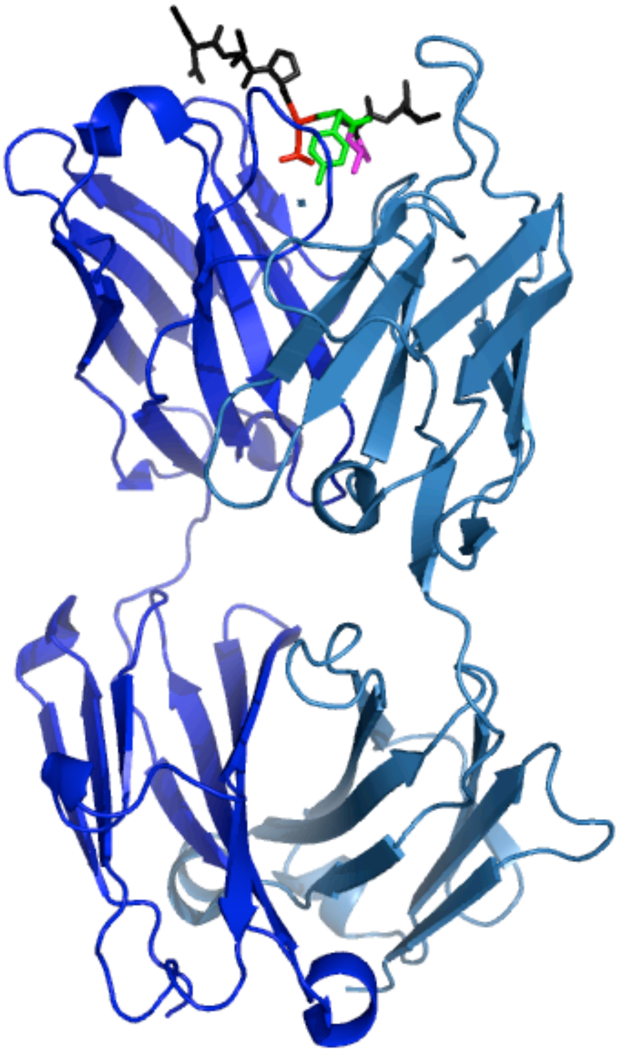

Figure S8. *Identification of residues involved in binding of the HA-tag to the anti-HA antibody Fab 26/9.* Structure of the entire tag/antibody complex (RDB code 1FRG). The HA tag is represented as black sticks, with residues D7, Y8 and A9 in red, green and magenta, respectively. These three residues are buried inside the interaction pocket of the antibody.

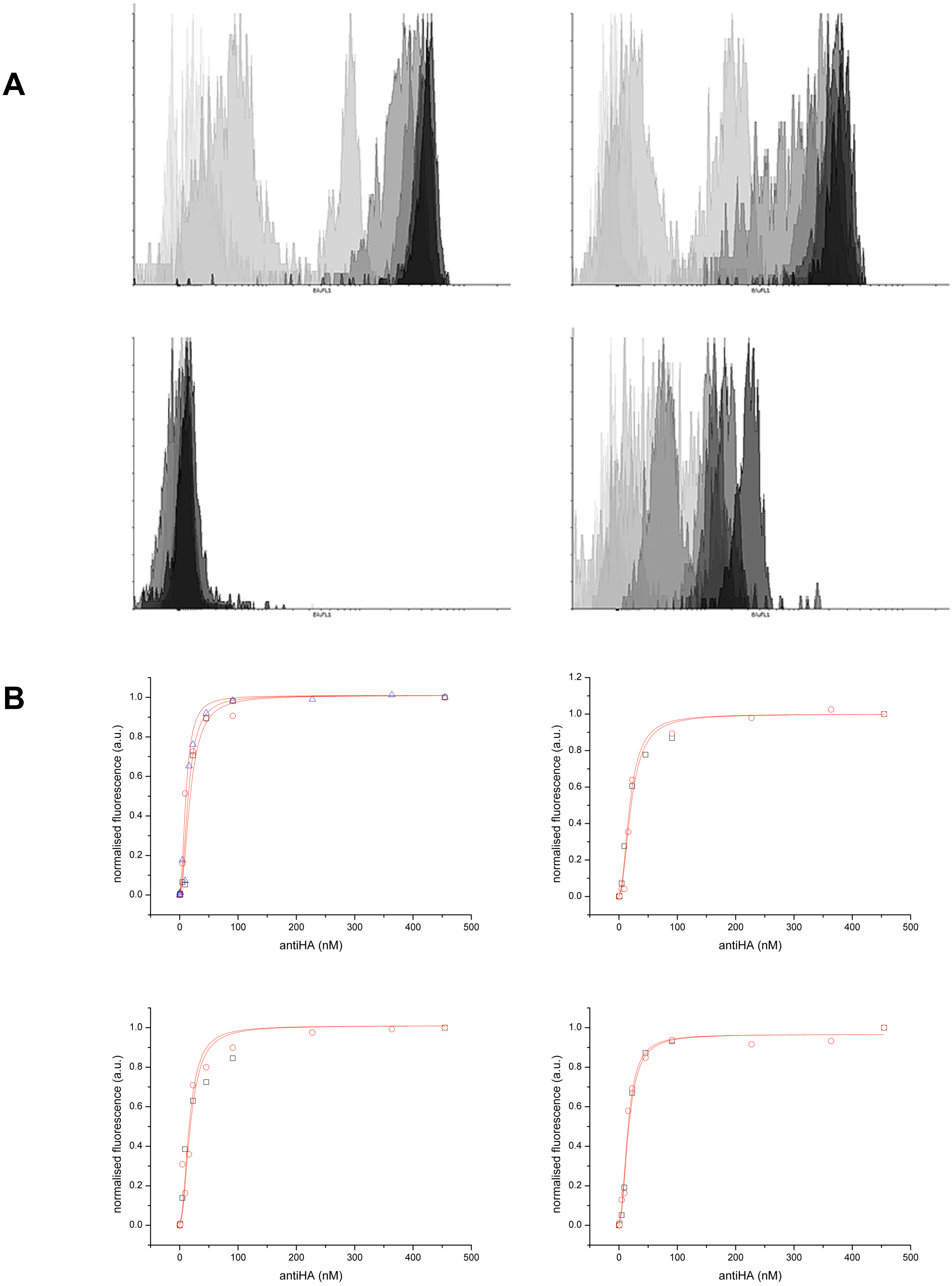

Figure S9. *Fluorescence histograms used for the determination of K_d on beads.* **(A)** Flow cytometry histograms representing from top to bottom: HA tag wt (top left), N7Y8S9 (top right), the negative control (no protein, bottom left) and T7K8L9 (bottom right). The gray gradient corresponds to the concentration of Alexa Fluor® 488-conjugate anti-HA antibody from 0.1 nM to 450 nM. **(B)** Reproducibility of the affinity assay on beads: HA tag wt (top left), N7Y8S9 (top right), N7Y8A9 (bottom left) and D7Y8S9 (bottom right).

Additional procedure for Figure 4

The visualization of PCR products on gel in Figure 4 was carried out using the following protocol: The PCR reaction (50 µL) contained DNA template, dNTPs (0.25 mM each), the forward and reverse oligonucleotides (0.2 µM each) and BioTaq DNA polymerase (4.5 U) in BioTaq buffer with 2 mM $MgCl_2$ (Bioline). The following protocol was used for 30 cycles: initial denaturation for 30 s at 95 °C; 30 cycles of denaturation for 30 s at 94 °C, annealing for 1 min at 48 °C and polymerization for 5 min at 72 °C.

NB. This PCR protocol was only used for the gel analysis of PCR products in Figure 4. See the procedure in the Experimental Section for the standard BeSD procedure involving ePCR.

References

Margulies M., Egholm M., Altman W.E., Attiya S., Bader J.S., Bemben L.A., Berka J., Braverman M.S., Chen Y.J., Chen Z. *et al.* (2005) *Nature,* **437**, 376-380.

Novak R., Zeng Y., Shuga J., Venugopalan G., Fletcher D.A., Smith M.T. and Mathies R.A. (2011) *Angewandte Chemie,* **123**, 410-415.
